# Genomic profile of the plants with pharmaceutical value

**DOI:** 10.1007/s13205-014-0218-9

**Published:** 2014-04-18

**Authors:** Saikat Gantait, Sandip Debnath, Md. Nasim Ali

**Affiliations:** 1 Department of Crop Science, Faculty of Agriculture, Universiti Putra Malaysia, 43400 Serdang, Selangor Malaysia; 2 Department of Biotechnology, Instrumentation and Environmental Science, Bidhan Chandra Krishi Viswavidyalaya, Mohanpur, 741252 West Bengal India; 3 Department of Genetics and Plant Breeding, Bidhan Chandra Krishi Viswavidyalaya, Mohanpur, 741252 West Bengal India; 4 Department of Agricultural Biotechnology, Faculty Centre for Integrated Rural Development and Management, School of Agriculture and Rural Development, Ramakrishna Mission Vivekananda University, Ramakrishna Mission Ashrama, Narendrapur, Kolkata, 700103 India

**Keywords:** AFLP, ESTs, Molecular marker, Polymerase chain reaction, RAPD, RFLP, SNP, SSR, DNA barcoding

## Abstract

There is an ample genetic diversity of plants with medicinal importance around the globe and this pool of genetic variation serves as the base for selection as well as for plant improvement. Thus, identification, characterization and documentation of the gene pool of medicinal plants are essential for this purpose. Genomic information of many a medicinal plant species has increased rapidly since the past decade and genetic resources available for domestication and improvement programs include genome sequencing, expressed sequence tags sequencing, transcript profiling, gene transmit, molecular markers in favor of mapping and breeding. In recent years, multiple endeavors have been undertaken for genomic characterization of medicinal plant species with the aid of molecular markers for sustainable utilization of gene pool, its conservation and future studies. Recent advancement in genomics is so fast that only some researches have been published till date and to a large extent documentation is restricted to electronic resources. Whole genome profiling of the identified medicinal plant species, carried out by several researchers, based on the DNA fingerprinting, is well documented in the present review. This review will facilitate preparing a database of the widely used, economically important medicinal plant species, based on their genomic organization.

## Introduction

In recent years, there has been a renewed awareness observed all over the world, concerning natural medicines that are extracted from plant parts. In this respect, 40 % or more of the pharmaceuticals used by the Western countries, at present, are the derivative of natural resources. Quite an impressive and substantial number of plant species have already been well described in detail, in *Ayurveda*, the original indigenous system of Indian medicine (Mukherjee and Wahile 2006). Amidst its diverse climatic zones, India has a wealth of medicinal herbs with immense diversity. The forests harbor a large number of plant species, but deforestation has been solely accountable for the rapid loss of the said natural wealth to such an extent that numerous important medicinal plants are now endangered and under the risk of extinction. Pharmaceutical companies rely mostly upon materials obtained from plants growing in their natural habitats that are being depleted in an alarming rate. It is well documented that, use of medicinal herbs and trees is increasing with a skyrocketing speed every day. A large-scale commercial utilization of these *Genuses* is speculated here and it calls for a proportionate involvement of modern day crop improvement methods for genetic betterment of the plants described earlier. It must be admitted that, modern day crop improvement is moving towards a new platform of genomics. The advantages of genomics have already been well harvested in different field crops. So, the present review has been documented and fashioned on the scope of genomics on genetic improvement of plants having medicinal value. There are several reports available on molecular markers of different medicinal plants. Genetic markers in plants consist of single-nucleotide polymorphisms (Hyten et al. 2010; Myles et al. 2010; Arai-Kichise et al. 2011; Barbazuk and Schnable 2011; Marroni et al. 2011) and microsatellites (SSR, simple sequence repeats; short tandem repeats) (Csencsics et al. 2010; Buehler et al. 2011; Delmas et al. 2011; Gardner et al. 2011; Michalczyk et al. 2011). These molecular markers are employed for genome mapping and the invention of disease-associated alleles, along with population mapping, and forensics, the analysis of germplasm, trait mapping, and marker-assisted selection in plant breeding and others (Appleby et al. 2009). Several assays have been developed for high-throughput genotyping in plants using these markers (Appleby et al. 2009; Jones et al. 2009). Genomic polymorphisms can also be wrapped up for validation of identity of species, devoid of prior sequence information, by means of sequence-independent array technology (Niu et al. 2011; Jayasinghe et al. 2009). Nonetheless, NGS is fast becoming the alternative technique for the characterization of genetic markers (Myles et al. 2010; Marroni et al. 2011; Elshire et al. 2011; Arai-Kichise et al. 2011; Deschamps and Campbell 2010) and the unambiguous detection of multiple alleles of homologous loci in polyploid plants (Griffin et al. 2011). Despite the escalating use of DNA sequence-based approaches, yet fingerprinting techniques persist to be exploited for genomic profiling of medicinal plants (Zhou et al. 2008; Kumar et al. 2007; Techen et al. 2010; Xue and Xue 2008; Qiu et al. 2009; Diao et al. 2009; Ruzicka et al. 2009; Tamhankar et al. 2009; Gupta et al. 2007; Huang et al. 2009; Li et al. 2009). A remarkable and unique progress is the utilization of real-time PCR for the quantitation of amplified markers (Xue and Xue 2008), which should facilitate the determination of the degree of adulterant contamination in primary samples.

## Impact of molecular marker techniques

Through the introduction of molecular markers, it is currently feasible to create straight inferences in relation to genetic divergence and inter-relationships amongst organisms at DNA level, devoid of the misleading environmental influences or imperfect pedigree accounts. Genetic assessment of plant populations and species, for taxonomic, evolutionary, and ecological research, has been immensely assisted from the advancement of a range of molecular marker systems. Even though every molecular marker system is derived from distinctive principles, yet their function is to expose the genome-wide variability. An evident difficulty that typically arises is as to how to select the apposite DNA marker amid the countless marker systems. Overall, the preference of a molecular marker system should be conciliated between consistency and simplicity of analysis, statistical rule, and assurance of exposing polymorphisms. Molecular systems are helpful in characterization of genetic divergence amongst several cultivars or species for the evaluation of genetic fidelity, identifying genes of commercial and agronomic interests, and enhancement via genetic transformation system. Su et al. (2008) reported the development of strain-specific sequence-characterized amplified region (SCAR) markers for strain detection and authentication of *Ganoderma lucidum*. Balasubramani et al. (2011) developed DNA markers from the genomic DNA through amplification and sequencing of the whole internal transcribed spacer region (ITS1, 5.8S rRNA and ITS2). They also established that the use of universal primers proved to be efficient and reliable in authenticating *Berberis* species. These primers are functional as a molecular pharmacognostic means in quality assessment of raw drugs. In an altogether different approach, Wang et al. (2012) employed the methylation-sensitive amplified polymorphism (MSAP) marker to appraise cytosine methylation difference in several regenerated plantlets and among organs of *Clivia miniata*. Wiriyakarun et al. (2013) reported the utilization of polymerase chain reaction restriction fragment length polymorphism (PCR–RFLP) to recognize plant origin of *Pueraria candollei*, *Butea superb* and *Mucuna collettii*. Nevertheless, to develop marker-assisted selection (MAS) breeding (for example in *Catharanthus roseus*) Chaudhary et al. (2013) reported the detection and mapping of QTLs, affecting pharmaceutical alkaloid contents in leaf and root. Some of the important achievements completed in medicinal plant species through molecular approaches are described in Tables 1, 2, and 3. Detection of genetic variation is also imperative for micropropagation and in vitro germplasm conservation to eradicate unwanted somaclonal variations. Somaclonal variations take place in reaction to the in vitro stresses and are manifested in the form of DNA methylation, chromosome reorganizations, and point mutations (Phillips et al. 1994). Therefore, a quality test for true-to-type planting material at an early stage of development is reflected to be helpful for in vitro culture. Molecular markers provide a significant tool to verify the genetic consistency and to test the true-to-type character of in vitro regenerated plants.

**Table 1:** Employment of RAPD markers in molecular characterization of medicinal plants

Medicinal plant species	Achievements	References
*Simmondsia chinensis* L. Schneider	Detection of genetic variability	Amarger and Mercier (1995)
*Afgekia sericea* Craib	Genetic diversity within and among Thai populations	Prathepha and Baimai (1999)
*Mentha* species	Assessment of genetic relationships in 11 accessions from six taxa of *Mentha* developed by CIMAP	Khanuja et al. (2000)
*Dioscorea floribunda*	Detection of genetic alteration in the post-cryo regenerated plants	Ahuja et al. (2002)
*Panax sikkimensis,P*. *pseudoginseng* and *P*. *quinquefolium*	Genetic and metabolomic demarcations	Mathur et al. (2003)
*Cymbopogon martinii*	Examination for genomic and expressed molecular diversity among the elite and popular cultivars	Sangwan et al. (2003)
*Boerhavia diffusa*	Assessment of genetic variability between accessions of different geographical origin within the Indian territory	Shukla et al. (2003)
*Droseraanglica* and *D. binata*	Verification of the clonal fidelity of two micropropagated plantlets	Kawiak and Łojkowska (2004)
*Azadirachta indica* A. Juss.	Assessment of genetic diversity and genetic relationships among 29 populations	Deshwal et al. (2005)
*Chlorophytum arundinaceum* Baker	Assessment of genetic fidelity of micropropagated plantlets	Lattoo et al. (2006)
*Lamiophlomis rotata*	Determination of genetic diversity and population structure employing a total of 188 individuals from eight natural populations	Liu et al. (2006)
*Mucuna prurien*s (L.) DC. (velvetbean)	Estimation of genetic diversity in 13 accessions	Padmesh et al. (2006)
*Typhonium trilobatum*	Identification genetic relationships in three species and evaluation of genetic variance within populations	Rout (2006)
*Simmondsia chinensis* L. Schneider	Identification of sex	Agrawal et al. (2007)
*Scutellaria baicalensis* Georgi (Huang-qin)	Assessment of genetic stability of the germplasm lines in long term in vitro maintained cultures	Alan et al. (2007)
*Pueraria montana* (Lour.) Merr. var. *lobata* and *Pueraria phaseoloides* (Roxb.) Benth.	Assessment of genetic variation of five *P. montana* var. *lobata* and 16 *P. phaseoloides* accessions	Heider et al. (2007)
*Dioscorea bulbifera* L.	Assessment of genetic fidelity of in vitro regenerants	Narula et al. (2007)
*Siraitia grosvenorii*	Assessment of genetic diversity in cultivars and wild accessions	Tang et al. (2007)
*Dictyospermum ovalifolium*	Assessment of genetic stability of in vitro grown plantlets	Chandrika et al. (2008)
*Tribulus terrestris*	Detection of genetic polymorphism in this medicinal herb collected from various geographical regions of India	Sarwat et al. (2008)
*Simmondsia chinensis* L. Schneider	A comparative study of genetic relationships among and within male and female genotypes	Sharma et al. (2009)
*Ginkgo biloba* L.	Sex type determination	Liao et al. (2009)
*Taxus wallichiana*	Assessment of genetic variation in nine natural populations from western part of the Himalayan ranges	Mohapatra et al. (2009)
*Atalantia monophylla*, *A. racemosa* and *A. wightii* (L.) DC.	Assessment of genetic diversity within and between populations	Ranade et al. (2009)
*Simmondsia chinensis* L. Schneider	Comparative assessment of marker assays for genetic diversity analysis	Bhardwaj et al. (2010)
*Bacopa monnieri* (L.)	Evaluation of genetic integrity in micropropagated plants and those of mother plants	Ceasar et al. (2010)
*Chlorophytum borivilianum* Sant. *et* Fernand	Assessment of genetic similarity of micropropagated plantlets with their mother plant	Kumar et al. (2010)
*Trollius*	Genetic diversity assessment	Li and Ding (2010)
*Chlorophytum borivilianum*	Assessment of the genetic stability of micropropagated and in vivo plant materials	Samantaray and Maiti (2010)
*Zingiber* *montanum* (Koenig) Link ex Dietr.	The genetic relatedness among 51 accessions, 14 species of the genus *Zingiber* and genetic variability of a clonally propagated species	Bua-in and Paisooksantivatana (2010)
*Vitex negundo* L.	Confirmation of the true-to-type nature of randomly selected micropropagated 2-year-old plant	Ahmad and Anis (2011)
*Swertia chirata*	Assessment of genetic fidelity of plantlets regenerated through somatic embryogenesis	Balaraju et al. (2011)
*Simmondsia chinensis* L. Schneider	Sex determination	Hosseini et al. (2011)
*Trigonella foenum*-*graecum*	Multilocus genotyping for detection of intraspecific variations	Kakani et al. (2011)
*Bacopa monnieri* (L.) Pennell	Assessment of genetic diversity in different accessions	Karthikeyan et al. (2011)
*Simmondsia chinensis* L. Schneider	Assessment of genetic fidelity of micropropagated plants	Kumar et al. (2011)
*Trichopus zeylanicus* subsp. *travancoricus*	Assessment of genetic fidelity of in vitro regenerants	Martin et al. (2011)
*Kaempferia galanga* L.	Molecular profiling of micropropagated plantlets	Mohanty et al. (2011)
*Aloe vera* L.	Assessment of genetic stability and instability of tissue culture-propagated plantlets	Rathore et al. (2011)
*Terminalia arjuna*, *T. bellerica* and *T. chebula*	Estimation of genetic diversity and evaluation of relatedness	Sarwat et al. (2008)
*Artemisia herba alba*	Genetic diversity of populations in central and north Saudi Arabia	Badr et al. (2012)
*Rauvolfia tetraphylla*	Assessment of DNA fingerprinting patterns among the micropropagated plants	Faisal et al. (2012)
*Mucuna pruriens* L.	Assessment of stability of among all the in vitro regenerated clones at the molecular level	Lahiri et al. (2012)
*Glycyrrhiza* *glabra*	Determination of genetic fidelity of plants obtained after conversion of alginate beads	Mehrotra et al. (2012)
*Nepenthes khasiana* Hook f.	Determination of genetic variation and gene flow estimation	Nongrum et al. (2012)
*Withania somnifera*	Assessment of variation among plants regenerated through indirect organogenesis	Rana et al. (2012)
*Panax quinquefolius* L.	Assessment of genetic relationships among and within populations	Schlag and McIntosh (2012)
*Bacopa monnieri* L.	Assessment of genetic variations among 15 accessions from Central India	Tripathi et al. (2012)
*Pleurotus* *citrinopileatus* Singer	Assessment of genetic diversity of the cultivars in China	Zhang et al. (2012)
*Vitex trifolia*	Establishment of genetic conformity of the in vitro regenerated plants	Ahmad et al. (2013)
*Ceropegia santapaui*	Assessment of DNA fingerprinting profile among the micropropagated plants	Chavan et al. (2014)
*Sapindus trifoliatus* L.	Estimation of genetic variability and population structure	Mahar et al. (2013)
*Picrorhiza kurroa* Royle ex Benth	Evaluation of genetic fidelity among in vitro regenerated plants	Rawat et al. (2013)
*Solanum trilobatum* L.	Assessment of genetic diversity across 14 accessions obtained from five South Indian states.	Shilpha et al. (2013)
*Murraya koenigii* (L.) Spreng	Genetic relationships among wild and cultivated accessions	Verma and Rana (2013)
*Viola pilosa*	Analysis of genetic stability of the in vitro grown plants	Soni and Kaur (2014)

**Table 2:** Employment of ISSR markers in molecular characterization of medicinal plants

Medicinal plant species	Achievements	References
*Lamiophlomis rotata*	Determination of genetic diversity and population structure employing a total of 188 individuals from eight natural populations	Liu et al. (2006)
*Swertia chirayita*	Analysis of genetic diversity of genotypes collected from the temperate Himalayas of India	Joshi and Dhawan (2007)
*Cunila* D. Royen ex L.	To examine the genetic diversity among the South American species	Agostini et al. (2008)
*Swertia chirata*	Evaluation of their clonal fidelity of plants raised through direct organogenesis	Kundu Chaudhuri et al. (2008)
*Tribulus terrestris*	Detection of genetic polymorphism in this medicinal herb collected from various geographical regions of India	Sarwat et al. (2008)
*Terminalia arjuna*, *T. bellerica* and *T. chebula*	Estimation of genetic diversity and evaluation of relatedness	Sarwat et al. (2008)
*Simmondsia chinensis* L. Schneider	Marker-assisted selection of male and female plants	Sharma et al. (2008)
*Dysosma pleiantha*	Assessment of genetic variation and genetic structure of species distributed in southeastern China	Zong et al. (2008)
*Allium ampeloprasum* L.	Determination of genetic integrity of micropropagated plantlets	Gantait et al. (2009)
*Thymus daenensis*	Detection of genetic polymorphism using 17 accessions collected from different geographic regions in Iran	Rahimmalek et al. (2009)
*Simmondsia chinensis* L. Schneider	A comparative study of genetic relationships among and within male and female genotypes	Sharma et al. (2009)
*Simmondsia chinensis* L. Schneider	Comparative assessment of marker assays for genetic diversity analysis	Bhardwaj et al. (2010)
*Aloe vera* L.	Assessment of genetic fidelity of micropropagated plants	Gantait et al. (2010a)
*Allium ampeloprasum* L.	Determination of genetic integrity in long-term micropropagated plantlets	Gantait et al. (2010b)
*Cannabissativa* L.	monitor the genetic stability of the micropropagated plants up to thirty passages in culture	Lata et al. (2010)
*Chlorophytum borivilianum* Sant. *et* Fernand	Assessment of genetic similarity of micropropagated plantlets with their mother plant	Kumar et al. (2010)
*Stevia rebaudiana Bert.*	Assessment of genetic fidelity of micropropagated plants	Das et al. (2011)
*Aloe vera* L.	Assessment of genetic similarity of micropropagated plantlets with their mother plant	Gantait et al. (2011)
*Simmondsia chinensis* L. Schneider	Assessment of genetic fidelity of micropropagated plants	Kumar et al. (2011)
*Aloe vera* L.	Assessment of genetic stability and instability of tissue culture-propagated plantlets	Rathore et al. (2011)
*Kaempferia galanga* L.	Molecular profiling of micropropagated plantlets	Mohanty et al. (2011)
*Sida* *species*	Assessment of degree of genetic diversity among different species	Thul et al. (2011)
*Astragalus membranaceus* (Fisch) *Bunge*	Development of sequence characterized amplified region (SCAR) markers	Yang et al. (2011)
*Rauvolfia tetraphylla*	Assessment of DNA fingerprinting patterns among the micropropagated plants	Faisal et al. (2012)
*Mucuna pruriens* L.	Assessment of stability of among all the in vitro regenerated clones at the molecular level	Lahiri et al. (2012)
*Glycyrrhiza* *glabra*	Determination of genetic fidelity of plants obtained after conversion of alginate beads	Mehrotra et al. (2012)
Two tetraploid species of *Hibiscus*	Determination of Genome synteny and evolution of allotetraploids	Satya et al. (2012)
*Sapindus trifoliatus* L.	Estimation of genetic variability and population structure	Mahar et al. (2013)
*Picrorhiza kurroa* Royle ex Benth	Evaluation of genetic fidelity among in vitro regenerated plants	Rawat et al. (2013)
*Solanum trilobatum* L.	Assessment of genetic diversity across 14 accessions obtained from five South Indian states	Shilpha et al. (2013)
*Balanites aegyptiaca*	Evaluation of clonal integrity of micropropagated plantlets chosen from a clonal collection	Varshney and Anis (2013)
*Murraya koenigii* (L.) Spreng	Genetic Relationships Among Wild and Cultivated Accessions	Verma and Rana (2013)
*Ceropegia santapaui*	Assessment of DNA fingerprinting profile among the micropropagated plants	Chavan et al. (2014)
*Viola pilosa*	Analysis of genetic stability of the in vitro grown plants	Soni and Kaur (2014)

**Table 3:** Employment of AFLP markers in molecular characterization of medicinal plants

Medicinal plant species	Achievements	References
*Dioscorea rotundata* and *D. cayenensis*	Assessment of genetic diversity	Mignouna et al. (1997)
*Azadirachta indica*	Assessment of genetic diversity	Singh et al. (1999)
*Datura* sp.	Assessment of genetic diversity	Mace et al. (1999)
*Moringa oliefera*	Determination of genetic variation	Muluvi et al. (1999)
*Cichorium* sp.	Diagnostic marker for endive and chicory	Kiers et al. (2000)
*Azadirachta indica*	Clonal fidelity in tissue culture raised plants	Singh et al. (2002)
*Actaea racemosa* L.	Identification and discrimination from three other closely related sympatric species	Zerega et al. (2002)
*Papaver* (section Oxytona), in vitro cell lines of *P. bracteatum*	Assessment species differentiation and genetic variability of in vitro-cultured plantlets	Carolan et al. (2002)
*Azadirachta indica* A. Juss.	Establishing clonal fidelity of tissue culture-raised plants	Singh et al. (2002)
*Solanum nigrum* complex	Assessment of genetic relationship between 14 genotypes of black nightshade	Jacoby et al. (2003)
*Echinacea purpurea*	Genetic mapping of via DNA fingerprinting of individual pollen	Aziz and Sauve (2008)
*Taxus wallichiana*	Assessment of genetic variation in nine natural populations from western part of the Himalayan ranges	Mohapatra et al. (2009)
*Carthamus tinctorius*	Inheritance and molecular marker analyses for HSYA trait	Zhang et al. (2009)
*Echinacea tennesseensis* (Beadle) Small	Phenotypic analyses during in vitro multiplication, storage, and acclimatization into soil	Moraes et al. (2011)
*Valeriana jatamansi* Jones	Assessment of genetic diversity and population structure in Western Himalaya, India	Rajkumar et al. (2011)

## Application of RAPD

The root of RAPD method is the differential PCR amplification of genomic DNA. It infers DNA polymorphisms generated through “rearrangements or deletions at or between oligonucleotide primer binding sites in the genome” via short random oligonucleotide sequences (mostly ten bases long) (Williams et al. 1991). It can be utilized across species by means of universal primers because this approach does not entail prior information of the genome under analysis. The chief shortcoming of this approach is that the profiling is contingent on the reaction circumstances that may fluctuate amid laboratories and since a number of distinct loci in the genome are amplified by each primer, profiles are incompetent to differentiate heterozygous from homozygous members (Bardakci 2001). Nevertheless, due to the swiftness and effectiveness of RAPD analysis, high-density genetic mapping in loads of medicinal plant species such as *Pueraria montana* (Heider et al. 2007), *Atalantia* species (Ranade et al. 2009), *Chlorophytum borivilianum* (Samantaray and Maiti 2010), *Swertia chirata* (Balaraju et al. 2011), *Withania somnifera* (L.) Dunal (Rana et al. 2012) have been achieved. In *Bacopa monnieri*, Ramesh et al. (2011) used a RAPD fingerprinting approach to evaluate the genetic stability of 19 different micropropagated clones with the mother plant (wild type). Even though this study illustrates the sustained use of RAPD, relative assessment of a variety of DNA fingerprinting techniques indicate that this technique is less reliable and more complicated to implement consistently in comparison to AFLP, SSR, and ISSR (Pejic et al. 1998; McGregor et al. 2000). The details of RAPD markers used in molecular assessment of medicinal plants are being included in Table 1.

## Application of ISSR

Several research groups used inter-simple sequence repeat (ISSR) as a better alternative DNA fingerprinting technique to appraise the genetic framework and diversity of wild and cultivated medicinal plants. Qiu et al. (2009) used ISSR markers to portray the genetic variation in wild and cultivated *Rhizoma corydalis*, a Chinese herbal medicine. In southern regions of China, the wild population of a medicinal plant species, *Corydalis yanhusuo* W.T. Wang ex Z.Y. Su et C.Y. Wu (Fumariaceae), has significantly been depleted and it has nearly been extinct from several other locations, owing to human interference along with environmental corrosion. These authors reported that the average within-population diversity of ISSR markers was superior in wild than in cultivated populations and suggested that the existing wild populations should be given a high precedence for in situ conservation as they possibly will function as reservoirs of genetic diversity in the species in the face of extinction (Qiu et al. 2009). Lata et al. (2010) reported that in vitro generated plants of *Cannabis sativa*, over 30 passages in culture and acclimatization in soil for 8 months, were genetically constant when assessed through ISSR markers and revealed a similar cannabinoid profile. The genetic variation amongst wild and cultivated populations of the Chinese medicinal plant *Coptis chinensis* (Ranunculaceae) was investigated using ISSR by Shi et al. (2008). They observed that the genetic diversity in wild and cultivated populations was largely similar but there was noteworthy genetic variation among the wild populations. Use of ISSR markers have been well documented in several other medicinal plants like *Swertia chirayita* (Joshi and Dhawan 2007), *Tribulus terrestris* (Sarwat et al. 2008), *Pleurotus citrinopileatus* (Zhang et al. 2012). The particulars of ISSR markers employed in molecular evaluation of such medicinal plants are described in Table 2.

## Application of AFLP

To triumph over the restraint of reproducibility coupled with RAPD, an amplified fragment-length polymorphisms (AFLP) technology (Vos et al. 1995) was developed. AFLP has several advantages to consider it applicable in the estimation of genetic diversity, genetic mapping, and tagging. AFLP has now developed into a favored approach as it unites the advantage of RFLP with the agility of PCR-based techniques by ligating primer recognition sequences to the restricted DNA and selective PCR amplification of restriction fragments via a restricted set of primers. The AFLP approach produces fingerprints of any DNA despite of its source, devoid of any previous information of DNA sequence. The primer pairs employed for AFLP typically generate 50–100 bands per assay. “Number of amplicons per AFLP assay is a function of the number selective nucleotides in the AFLP primer combination, the selective nucleotide motif, GC content and physical genome size and complexity”. Most AFLP fragments match up to distinctive locations on the genome and, therefore, can be used as pointer in genetic and physical mapping. The system can be used to differentiate strongly associated individuals at the sub-species level (Althoff et al. 2007) and can map genes too. Zerega et al. (2002) examined the use of AFLP as an analytical means of identifying *Actaea racemosa* from three other closely related sympatric species. A total number of 262 AFLP markers were generated, and one unique fingerprint was identified for *A. racemosa*, whereas two, six, and eight unique fingerprints were identified for the closely related species *A. pachypoda*, *A. cordifolia*, and *A. podocarpa*, respectively. Two commercial black cohosh products were also subjected to AFLP analysis and shown to contain only *A. racemosa*. The results of their study suggested that AFLP analysis may offer a useful method for quality control in the botanical dietary supplements industry. Applications for AFLP in plant mapping include genetic mapping of the pollen genome of *Echinacea purpurea* ‘Magnus’ (purple coneflower) which was amplified by a modified primer extension pre-amplification (PEP) procedure subsequently AFLP analyses of individual pollen grains (Aziz and Sauve 2008).

Using AFLP, He et al. (2009) investigated the population structure and genetic diversity of wild and cultivated populations of *Magnolia officinalis* subsp. *biloba* (Magnoliaceae) Plant. Principal coordinates analysis of AFLP data did not discriminate between wild and cultivated populations, which led the authors to conclude that alleles from the wild population were maintained in the cultivated gene pool. A sole report has been published in recent past by Agarwal et al. (2011) on development of sex-linked AFLP markers in jojoba (*Simmondsia chinensis*). Singh et al. (2002) reported application of AFLP markers for ascertaining clonal fidelity in tissue culture-raised progenies of a medicinally important plant, *Azadirachta indica*. AFLP markers are now being routinely employed for assessment of genetic variation in economically important plant species including chichory (Kiers et al. 2000), *Withania* sp. (Negi et al. 2000) (Table 3).

## Use of microsatellite markers

“Microsatellite or short tandem repeats or simple sequences repeats (SSR) are monotonous repetitions of very short (one to five) nucleotide motifs, which occur as interspersed repetitive elements in all eukaryotic genomes” (Tautz and Renz 1984). However, strand slippage during DNA replication, where the repeats allow matching through excision or addition of repeats, results in disparity in the number of tandemly repeated units (Schlotterer and Tautz 1992). As slippage in replication is more expected than point mutations, microsatellite loci tend to be hypervariable. Microsatellite assays explain wide inter-individual length polymorphisms during PCR analysis of unique loci using discriminatory primers sets. High level of polymorphism and their co-dominant nature have made SSRs ideal markers for studying genetic diversity in plants (Plaschke et al. 1995). Hon et al. (2003) reported the efficiency of SSR markers in genetic authentication of two *Panax* species. Later on, Qin et al. (2005) employed SSR markers to identify and differentiate American ginseng and Oriental ginseng, cultivated and wild American ginseng. Huang et al. (2009) developed eight polymorphic microsatellite loci for the Chinese medicinal plant *Artemisia annua* L. (Asteraceae), useful for investigating the genetic diversity, genetic structure and gene flow within populations. Li et al. (2009) isolated polymorphic microsatellite loci and characterized them from an AC-enriched genomic library of *Akebia trifoliate* ssp. *australis*. A robust set of 17 polymorphic EST–SSRs were developed and used for evaluating 20 turmeric accessions (Siju et al. 2010). Eleven microsatellite markers were isolated and characterized from enriched genomic libraries of *Channa argus* (Gul et al. 2010). Rahimmalek et al. (2011) developed and characterized SSR markers for the first time from the genome of yarrow (*Achillea millefoilum* L.), using the slightly modified Hamilton protocol to allow the selection of desired genotypes rather than phenotypes and hence to accelerate the breeding programs. Microsatellite (GTG)_5_ were used to assess DNA polymorphism and genetic diversity in *Allium ampeloprasum* (Guenaoui et al. 2013). Likewise, Zhou et al. (2012) used 10 microsatellite (SSR) loci to investigate genetic diversity and differentiation in 16 natural populations of *Saruma henryi* Oliv. Use of cross-species SSR markers was reported in genetic diversity analysis of synthetic interspecific hybrid of *Hibiscus* which revealed a closer association of diploid genomes and high variability of tetraploid genomes (Satya et al. 2012). Most recently, Katoch et al. (2013) used DNA-based molecular marker techniques, viz. simple sequence repeats (SSR) and cytochrome P-450 markers to estimate genetic diversity in *Picrorhiza kurrooa*.

## Use of single nucleotide polymorphism (SNPs)

“Single nucleotide variations in genome sequence of individuals of a population are known as SNPs”. They comprise the most copious molecular markers in the genome and are extensively distributed all through genomes even though their occurrence and allocation vary between species. Maize has 1 SNP per 60–120 bp (Ching et al. 2002). The SNPs are generally more ubiquitous in the non-coding regions of the genome. An SNP, within the coding regions, is either non-synonymous and results in an amino acid sequence alteration (Sunyaev et al. 1999), or it is synonymous and fail to modify the amino acid sequence. Synonymous changes can modify mRNA splicing, ensuing in phenotypic variations (Richard and Beckman 1995).

Advancement in sequencing technology and accessibility of an escalating number of expressed sequence tags (EST) sequences has made direct analysis of genetic variation at the DNA sequence level achievable (Buetow et al. 1999; Soleimani et al. 2003). “Prevalence of SNP genotyping assays are based on one or two of the following molecular mechanisms: allele specific hybridization, primer extension, oligonucleotide ligation and persistent cleavage” (Sobrino et al. 2005). High-throughput genotyping methods counting DNA chips, allele-specific PCR, and primer extension approaches make SNPs especially attractive as genetic markers. They are apposite for automation and are employed for a variety of purposes, including rapid detection of crop cultivars and construction of ultra high-density genetic maps. Ginger (*Zingiberofficinale* Rosc), a well-known plant for its medicinal applications, was successfully applied for the development of high-throughput methods for the detection of SNPs and small indels (insertion/deletion). In that study, 64026 SNP sites and 7034 indel polymorphisms with frequency of 0.84 SNPs/100 bp were found and among the three tissues from which the EST libraries were generated, rhizomes had high frequency of 1.08 SNPs/indels per 100 bp whereas the leaves had lowest frequency of 0.63 per 100 bp and root showed relative frequency of 0.82/100 bp (Chandrasekar et al. 2009). Work has been done for authentication of medicinal plants by SNP-based Multiplex PCR, where highly variable intergenic spacer and intron regions from nuclear and cytoplasmic DNA have been used for species identification (Lee et al. 2012). Noncoding internal transcribed spacers (ITSs) located in 18S-5.8S-26S, and 5S ribosomal RNA genes (rDNAs) were the target region for this purpose. In this context, noncoding regions from two cytoplasmic DNA, chloroplast DNA (*trnT*-F intergenic spacer region), and mitochondrial DNA (fourth intron region of *nad7* gene) were also successfully applied for the proper identification of medicinal plants and SNP sites obtained from the amplification of intergenic spacer and intron regions were properly utilized for the verification of medicinal plants in species level using multiplex PCR. *Dendrobiumofficinale* is used as a crude drug in traditional Chinese medicine (TCM) having tonic efficacy. For crude drug quality control and authentication of different populations the sequences from its chloroplast, nuclear, and mitochondrial genes and the method of genomic DNA (gDNA) suppression subtraction hybridization (SSH) were successfully used. In that experiment, six populations were authenticated successfully by nine SNP sites and six pairs of diagnostic primers. Amplification refractory mutation system (ARMS) was also designed to identify six populations on the basis of SNPs (Ding et al. 2008).

## Expressed sequence tags of medicinal plants

Expressed sequence tags (ESTs), identified by the single-pass sequencing of randomly selected clones from the cDNA library, are molecular tools that are reasonably useful in defining an expressed gene, and also specify the profusion of transcripts. Large-scale EST databases offer a multitude of information concerning the complexities of gene expression patterns, the functions of transcripts, and the development of SNPs (Yang et al. 2004). In plant, large-scale EST databases have been recognized, and an array of ESTs procured from different tissues, developmental stages, and stress-treated cDNA libraries have been compared with model plants and crops. In most of the medicinal plants, nevertheless, the complete genome and draft sequences are yet to be established. Consequently, EST assay represents the most rational system for the study of the genome of the plants with medicinal importance; hence, several attempts have been made in this aspect by a number of research groups. Monoterpene indole alkaloid (MIA) pathway genes were identified from random sequencing of *C. roseus* cDNA library which revealed 3,655 unique ESTs, comprising 1,142 clusters and 2,513 single tons. A number of novel MIA pathway candidate genes were recognized by the cloning and functional characterization of loganic acid *O*-methyletransferase implicated in secologanin biosynthesis. Biochemical pathway, for instance, triterpene biosynthesis was also identified and its metabolite analysis revealed localization of oleanane-type triterpenes exclusively to the cuticular wax layer. The results illuminated biochemical specialization of *Catheranthus* leaf epidermis for the production of multiple classes of metabolites (Murata et al. 2008). Also, jasmonate-induced changes on the transcripts and alkaloid profiles of tobacco BY-2 and *C. roseus* cell cultures have been monitored through similar approach (Rischer et al. 2006; Goossens et al. 2003). ESTs analogous to 40 enzymes involved in the conversion of sucrose to sanguinarine were identified from elicitor-induced cell culture of *Papaver somniferum*. Significant enhancement in the level of RNA was observed in case of elicitate cell culture as compared to control and the identified metabolites were sanguinarin, dihydrosanguinarine, methoxylated derivatives, dihydrochelirubine and chelibine and the identified alkaloid pathway intermediates were *N*-methylcoclaurine, *N*-methylestylopine, and protopine (Zulak et al. 2005). Likewise, cDNA library of *Artimisia annua* glandular trichome revealed the occurrence of scores of ESTs implicated in isoprenoid biosynthesis for instance enzymes from the methylerythritol phosphate pathway and the mevalonate pathway, amorpha-4, 11-diene synthase and other sesquiterpene synthase, monoterpene synthases and 2-cDNAs revealing prominent resemblance to germacrene-A-synthases (Berteaa et al. 2006). Siju et al. (2010) used ESTs from turmeric (*Curcuma longa* L.) for the screening of type and frequency of Class I (hypervariable) simple sequence repeats (SSRs). Recently, to identify rhizome-enriched genes and genes encoding specialized metabolism enzymes and pathway regulators, Koo et al. (2013) evaluated an assembled collection of ESTs from eight different ginger and turmeric tissues. In *Panax ginseng*, Kim et al. (2006) found that 2,896 cDNA clones represent 1,576 unique sequences, consisting of 1,167 singletons and 409 contig sequences. The ESTs referenced in their report were the first transcriptomes in a leaf from a half-shade ginseng plant. The majority of the identified transcripts were found to be genes related with energy, metabolism, subcellular localization, protein synthesis and transport. Xie et al. (2008) built an EST database from four basil (*Ocimum basilicum* L.) lines with distinct product profiles providing the sequence foundation required for comparative proteomic studies. Cloutier et al. (2009) exploited EST to develop SSRs for genomic assessment of flax (*Linum usitatissimum* L.). Sathiyamoorthy et al. (2010) obtained a total of 6,757 ESTs from cDNA libraries in *Panax ginseng* C. A. Meyer. This EST dataset provides a wide outlook of the genes expressed in hairy roots, 14-year root and 4-year root. The dataset contains more than 1,365 EST sequences related to plant secondary metabolism and 745 sequences related to stresses. Li et al. (2010) used the 454 GS FLX platform and Titanium regents to produce a substantial EST dataset from the vegetative organs of *Glycyrrhiza uralensis*. Based on the EST analysis, novel candidate genes related to the secondary metabolite pathway of glycyrrhizin, including novel genes encoding cytochrome P450 s and glycosyltransferases, were found. However, there are a few and limited genomic resources available for *Picrorhiza* (Bantawa et al. 2012) in spite of its immense medicinal importance. A total of 728 ESTs of *P. kurroa* and 27 of *P. scrophulariflora* have been deposited at NCBI (www.ncbi.nlm.nih.gov). Kawoosa et al. (2010) have identified two regulatory genes of terpenoid metabolism, namely- 3-hydroxy-3-methylglutaryl coenzyme A reductase (pkhmgr) and 1-deoxy-d-xylulose-5-25 phosphate synthase (pkdxs) from *P. kurroa*. An account of hundreds of genes implicated not only in alkaloid biosynthesis but also in plant secondary metabolism. Subsequently, large-scale functional analysis of genes from this inventory, potentially involved in plant secondary metabolism, was carried out. This comprises isolation, introduction, and functional assay of full-length open reading frames in transgenic plant cells. Tools to enhance and accelerate functional analysis of candidate genes in transgenic plant cells with reporter gene constructs, transient protoplast expression assays, and microarray facilities, have been designed and their uses validated.

## Microchip based genomic profiling

High-throughput and automated genomic techniques have been proven to speed up modern day research for generating large number of data. DNA microarray is a arrayed series of thousands of microscopic spots of DNA oligonucleotides, called features, each containing picomoles (10–12 mol) of a specific DNA sequence, known as probes (or reporters). It can be used to measure changes in expression levels, detection of SNPs, to genotype or resequence mutant genomes (Hao et al. 2010). This techniques was successfully applied to identify toxic traditional Chinese medicinal plants, where species- specific oligonucleotide probes were obtained from the 5S rRNAgene of *Aconitum carmichaeli* Debx., *A. kusnezoffi* Reichb., *Alocasia macrorrhiza* (Linn.) Schott, *Croton tiglium* L., *Datura inoxia* Mill., *D. metel* L., *D. tatula* L., *Dysosma pleiantha* (Hance) Woodson, *D. versipellis* (Hance) M. Cheng ex Ying, *Euphorbia kansui* L., *Hyoscyamus niger* L., *Pinellia cordata* N.e. Brown, *P. pedatisecta* Schott, *P. ternata* (Thunb.) Breit., *Rhododendron molle* (Blum) G. Don, *Strychnos nux*-*vomica* L., *Typhonium divaricatum* (Linn.) Decne., and *T. giganteum* Engl., and the leucine transfer RNA gene of *A. pendulum* Busch and *Stellera chamaejasme* L. (Carles et al. 2005). This is a rapid tool for quality control and safety monitoring of herbal pharmaceuticals and neutraceuticals. There are other reports on the use of microarray for high throughput identification of the plant resource of commercial FDSH [*Fengdu Shihu* (*Dendrobium officinale* Kimura et Migo)] (Sze et al. 2008), identification of various *Panax* plants and drugs (Zhu et al. 2008), genotyping, and identification of the origin of various species of *Fritillaria* L. at molecular level (Tsoi et al. 2003).

## DNA barcoding

DNA barcoding is the application of molecular phylogeny where, the species of an individual organism is signatured using small sections of chloroplast/nuclear/mitochondrial DNA. A DNA barcode is a potential tool for taxonomic identification that employs short, standardized DNA sequences (mostly 400–800 bp) present universally in target lineages and has ample sequence variation to differentiate among species of a specific organism. This offers a quick and precise system for definite species identification through/via adequate sequence variation amongst species and little intraspecific variation. The universally accepted genes for plant DNA barcoding are of plastid origin. Twenty-five years following the publication of the first complete sequence of a chloroplast genome by Shinozaki et al. (1986); several scientists have used next-generation sequencing technology to sequence the complete chloroplast genomes from quite a few number of plants (Parks et al. 2009) (Doorduin et al. 2011). Polymorphism of chloroplast DNA particularly trnK, matK, and intergenic trnL–trnF regions have been exploited to learn the phylogeny of several plants (Kress et al. 2005; Selvaraj et al. 2008). However, *Buxus* (Buxaceae), *Chloranthus* (Chloranthaceae), *Dioscorea* (Dioscoreaceae), and *Illicium* (Schisandraceae) have comparatively strong chloroplast genome information that can be tapped (Hansen et al. 2007). Hao et al. (2009) showed the evolutionary patterns of gene sequence divergence from the medicinal genus *Taxus* L., encoding paclitaxel biosynthetic enzymes taxadiene synthase (TS) and 10-deacetyl- baccatin III-10 beta-*O*-acetyltransferase (DBAT). According to Yao et al. (2009), the psbA-trnH spacer regions were effective barcodes for species of *Dendrobium* Sw. This technique was used for the first time to discriminate the Polygonaceae in *Chinese Pharmacopoeia* and their adulterants (Song et al. 2009). Chen et al. (2010) compared seven candidate DNA barcodes (psbA-trnH, matK, rbcL, rpoC1, ycf5, ITS2, and ITS) from medicinal plant species. Gao et al. (2010) showed ITS2 sequences to show considerable variation at the genus and species level within the family Fabaceae.

## Conclusion: future of medicinal plant genomics

Molecular techniques have been well proven for authentication of medicinal plants based on phylogenic variation signatured on chloroplast and nuclear DNA regions. DNA markers are the powerful tool for the characterization of sample homogeneity and detection of adulterants and thus assure quality control in medicinal plant research as well as in the production, clinical use, and forensic examination of herbal medicines for their safest use. So, there is always an immediate need to scrutinize the genomic tools in detail so as to harvest the advantages of genomics in total for the broad use of medicinal plant genome. A comprehensive study and research is a demand for all-round use of genomics for authentic and safe use of botanical products and medicinal plants. With the advent of new DNA sequencing platforms that achieve an ever-increasing degree of speed, coverage, and sharply decreasing costs for obtaining the data, we can expect to see many genome libraries, SNP chips and, ultimately, a complete genome sequencing (of plastid) effort. More recently, marker systems of various types have been developed and these have increased our knowledge of the population structure of various medicinal plant populations. The more recent sequencing of the nuclear and chloroplast genomes will allow the development of additional tools for studying not only the population structure but also the genetic basis of various traits and their inheritance. Genome sequencing projects may be initiated vividly for all the plants having medicinal use. In this regard, sequence databases with all the bioinformatics tools should be made available for public use by different nations; as such platforms are only available for rice and some other crop plants.

## Abbreviations

ESTs: Expressed sequence tags
RFLP: Restriction fragment length polymorphism
RAPD: Randomly amplified polymorphic DNA
AFLP: Amplified fragment length polymorphism
SSR: Simple sequence repeat
ISSR: Inter simple sequence repeat
SNP: Single-nucleotide polymorphism
UPGMA: Unweighted pair group method arithmetic average
